# Effectiveness of Digital Storytelling and Immersive Technology Interventions in Reducing Preoperative Anxiety Among Children: Systematic Review and Meta-Analysis

**DOI:** 10.2196/92880

**Published:** 2026-08-05

**Authors:** Fatimah Abdulaziz Almuqad, Ghaida Talea Alsaedi, Taif Abdulwahab Alahmadi, Sulafah Ibrahim Alalawi, Redwan Mohammed Mulla, Renad Marzouq Almohammadi, Maher Abulfaraj

**Affiliations:** 1 College of Medicine, Al-Rayan Colleges Al-Madinah Al-Munawwarah Saudi Arabia; 2 College of Medicine, Taibah University Al-Madinah Al-Munawwarah Saudi Arabia; 3 Pediatric Department, College of Medicine, Taibah University Al-Madinah Al-Munawwarah Saudi Arabia

**Keywords:** preoperative anxiety, children, virtual reality, immersive technology, digital storytelling

## Abstract

**Background:**

Preoperative anxiety affects an estimated 50% to 70% of children undergoing surgery and is associated with poorer perioperative cooperation, increased postoperative distress, and delayed recovery. Digital storytelling and immersive technologies, including virtual reality and augmented reality, have emerged as child-centered preparation tools to improve the preoperative experience.

**Objective:**

This systematic review and meta-analysis aimed to synthesize and critically appraise the available evidence on the effects of these interventions in reducing preoperative anxiety among pediatric patients undergoing elective surgery.

**Methods:**

A systematic search was conducted in PubMed, Scopus, Web of Science, and the Cochrane Library for studies published between January 2015 and June 2025. Randomized controlled trials and quasi-experimental studies evaluating digital storytelling (eg, narrative-based apps or videos) or immersive technologies (eg, virtual reality or augmented reality) were included. Preoperative anxiety was assessed using validated measures such as the Modified Yale Preoperative Anxiety Scale, State-Trait Anxiety Inventory, or Visual Analog Scale. Further, 2 reviewers independently screened studies and extracted data. Risk of bias was assessed using the Cochrane Risk of Bias tool version 2. A random-effects meta-analysis was performed in R using standardized mean differences.

**Results:**

A total of 379 records were identified, and 13 studies met the inclusion criteria. Most studies reported lower preoperative anxiety in intervention groups compared with standard care or conventional preparation. Further, 9 studies provided sufficient comparable data for meta-analysis, showing a significant pooled reduction in anxiety favoring digital interventions (standardized mean difference=−0.67, 95% CI −0.93 to −0.41), with substantial heterogeneity (*I*^2^=79.3%, *P*<.001). Overall risk of bias was high in most trials, primarily due to deviations from intended interventions and limitations in blinding.

**Conclusions:**

This review suggests a trend toward reduced preoperative anxiety among pediatric patients receiving digital storytelling and immersive technology–based interventions. However, the certainty of evidence is limited by substantial heterogeneity, the small number of studies included in the meta-analysis, variability in outcome measures, and overall study quality. Therefore, the pooled estimate should be interpreted with caution. Further high-quality studies using standardized outcomes and robust methodology are needed, including evaluation of parental anxiety and longer-term postoperative effects.

**Trial Registration:**

PROSPERO CRD420251111293; https://www.crd.york.ac.uk/PROSPERO/view/CRD420251111293

## Introduction

Preoperative anxiety is a common and often expected emotional response among children facing surgery, and it can arise at any point before the procedure [1]. It affects an estimated 50% to 70% of pediatric patients and represents a major concern in pediatric anesthesia and perioperative care [2]. High levels of anxiety can negatively influence cooperation with medical staff and increase perioperative distress. From a physiological perspective, heightened anxiety activates the stress response system, including increased sympathetic nervous system activity and hypothalamic-pituitary-adrenal axis activation, which may contribute to elevated heart rate, cortisol release, and heightened pain perception. These responses have been associated with greater postoperative pain and increased risk of emergence delirium [3,4].

From a psychological and behavioral perspective, anxiety may impair coping ability, increase anticipatory fear, and reduce a child’s capacity to engage with medical procedures, which can lead to poorer perioperative cooperation and increased distress during anesthesia induction. In addition, negative perioperative experiences may reinforce maladaptive fear responses and contribute to postoperative behavioral changes, such as sleep disturbances, separation anxiety, or regression, which may persist beyond hospitalization [3,4]. These interconnected physiological and psychological pathways highlight the clinical importance of identifying effective and developmentally appropriate approaches to reduce anxiety before surgery.

Traditional strategies for reducing pediatric preoperative anxiety include pharmacological approaches (eg, benzodiazepine premedication) as well as nonpharmacological interventions such as preoperative education, hospital tours, behavioral preparation programs, child life specialist support, role-play, and distraction techniques [2,5]. Although these approaches can be beneficial, many require additional staffing and time, may not be consistently available across institutions, and can be challenging to implement in busy or resource-limited settings [6]. As a result, there is ongoing interest in scalable, engaging, and noninvasive preparation methods that can be delivered efficiently and tailored to children’s developmental needs.

In recent years, digital storytelling and immersive technologies have emerged as promising alternatives for pediatric preoperative preparation. In this review, digital storytelling refers to narrative-based preparation delivered through mobile or tablet apps, interactive stories, animated educational content, or video-based formats that explain the surgical journey in a child-friendly manner [7]. In contrast, immersive technologies include platforms such as virtual reality (VR) and augmented reality (AR) that provide interactive and immersive simulated environments, often through head-mounted displays or AR-enabled devices. These interventions may reduce anxiety through several complementary mechanisms, including attentional distraction, increased engagement, improved procedural understanding, and enhanced coping through familiarization with the hospital environment [8].

Previous systematic reviews and meta-analyses have reported beneficial effects of VR-based interventions on anxiety and related perioperative outcomes in pediatric settings; however, these reviews often focus predominantly on VR and may not fully capture newer narrative-based digital storytelling tools or more recent immersive applications [9,10].

Despite increasing research interest, the available evidence remains heterogeneous in intervention type, delivery mode, timing of exposure, surgical populations, and anxiety measurement tools. In addition, the overall certainty of evidence is influenced by variability in study quality and reporting. Therefore, this systematic review and meta-analysis aimed to synthesize and critically appraise the available evidence on the effects of digital storytelling and immersive technology–based interventions (including VR, AR, and app-based storytelling tools) in reducing preoperative anxiety among pediatric patients undergoing elective surgery, compared with standard care or conventional preparation methods.

## Methods

### Study Design

This study was a systematic review and meta-analysis designed to synthesize and critically appraise the available evidence on the effects of digital storytelling and immersive technology–based interventions on preoperative anxiety in pediatric patients undergoing elective surgery. The review was structured according to the PICOS (Population, Intervention, Comparison, Outcomes, and Study) framework: the population comprised pediatric patients (≤18 years) undergoing elective surgical procedures; the interventions included digital storytelling and immersive technologies such as mobile or tablet-based apps, VR, AR, and related digital preparation tools; comparators consisted of standard care or conventional preoperative preparation methods (eg, routine verbal or written information); outcomes focused on quantitatively measured preoperative anxiety using validated instruments; and eligible study designs included randomized controlled trials (RCTs) and quasi-experimental comparative studies. The review was conducted in accordance with the *Cochrane Handbook for Systematic Reviews of Interventions* and reported following the PRISMA (Preferred Reporting Items for Systematic Reviews and Meta-Analyses) 2020 guidelines [11,12]. The review protocol was prospectively registered in PROSPERO (CRD420251111293).

### Search Strategy

A comprehensive electronic search was performed in 4 databases: PubMed, Scopus, Web of Science, and the Cochrane Library. Searches were developed using a combination of free-text keywords related to the population (child, pediatric, or pediatric), the outcome (preoperative anxiety, surgical anxiety, or procedure-related anxiety), and the intervention (digital storytelling, interactive story, animated book, VR, AR, mobile or tablet apps, and video-based education).

These concepts were combined using Boolean operators (AND/OR) and adapted to the syntax and indexing requirements of each database. The search was restricted to studies published between January 2015 and June 2025. The PROSPERO registration (CRD420251111293) reflects the initial planned search period up to January 2025; however, an updated search was conducted in June 2025 before final analysis to ensure inclusion of the most recent evidence. The search was limited to English-language publications where applicable. In addition, the reference lists of included studies and relevant reviews were manually screened to identify any additional eligible papers. The complete database-specific search strategies are provided in Multimedia Appendix 1.

### Eligibility Criteria

Studies were eligible for inclusion if they met the following criteria:

- Population: pediatric participants undergoing elective surgical procedures. Although the review focus was children, studies were included if the participants were predominantly pediatric (≤18 years) or if pediatric data were extractable.
- Intervention: any digital or immersive preoperative preparation intervention delivered to the child, including (1) immersive technologies, such as VR, AR, or mixed reality (MR), and/or (2) digital storytelling interventions, such as narrative-based educational content delivered through mobile or tablet apps, interactive story formats, or video-based preparation tools. Studies in which the intervention combined digital and nondigital components (eg, verbal instruction plus a VR tool) were eligible only when the digital or immersive component was the primary active element intended to reduce anxiety.
- Comparator: standard care or conventional preparation methods, including routine verbal information, written materials, or usual preoperative practice.
- Outcomes: studies were required to report quantitative preoperative anxiety outcomes assessed using validated instruments such as the Modified Yale Preoperative Anxiety Scale (mYPAS), State-Trait Anxiety Inventory (STAI), Visual Analog Scale (VAS), or comparable validated measures.
- Study design: RCTs and quasi-experimental comparative studies.
- Publication characteristics: studies published in English with available full text.

Studies were excluded if they (1) included adult-only samples without extractable pediatric results; (2) did not report quantitative preoperative anxiety outcomes; or (3) used noncomparative publication formats, such as reviews, editorials, conference abstracts, protocols, or case reports.

### Definition of Digital Storytelling and Immersive Technology Interventions

For this review, digital or immersive interventions were defined as preoperative preparation tools delivered through digital media or immersive platforms with the goal of reducing anxiety by improving engagement, familiarization, distraction, or coping before surgery.

Digital storytelling interventions were defined as narrative-based educational content delivered via digital devices (eg, smartphones, tablets, computers, or hospital screens). These interventions included story-driven or character-guided formats such as interactive stories, animated storybooks, app-based storytelling programs, or video-based educational storytelling designed to explain the surgical journey in a developmentally appropriate manner [13].

Immersive technology interventions were defined as interventions using immersive or interactive environments, including VR, AR, or MR, typically delivered through a head-mounted display or interactive AR-enabled devices. These interventions commonly provided simulated exposure to perioperative environments (eg, operating room tours) or interactive distraction content aimed at reducing anticipatory distress [7].

Some interventions combined digital components with conventional nondigital elements (eg, routine verbal explanations alongside a digital tool). In such cases, studies were considered eligible when the digital or immersive component represented the primary active element of the intervention, was delivered directly to the child preoperatively, and the study design allowed comparison with a control group receiving standard care or conventional preparation alone.

### Study Selection and Screening

All search results were imported into Rayyan for initial screening and duplicate removal [14]. A total of 2 reviewers independently screened titles and abstracts for relevance. Full-text reviews were conducted for all studies that met the inclusion criteria or were deemed potentially eligible. Disagreements at any stage were resolved through discussion or by consulting a third reviewer to reach a consensus. Interrater agreement between the 2 reviewers during title and abstract screening was high (Cohen κ=0.82). Agreement at the full-text screening stage was also high (κ=0.86). Discrepancies were resolved through discussion or consultation with a third reviewer.

### Quality Assessment

The risk of bias of included RCTs was assessed using the Cochrane Risk of Bias tool version 2 (RoB 2) [15]. RoB 2 evaluates potential bias across five domains: (D1) bias arising from the randomization process, (D2) bias due to deviations from intended interventions, (D3) bias due to missing outcome data, (D4) bias in measurement of the outcome, and (D5) bias in selection of the reported result. Each domain was rated as low risk of bias, some concerns, or high risk of bias, and an overall risk-of-bias judgment was assigned for each study based on the RoB 2 algorithm. Further, 2 reviewers independently conducted the assessments, and disagreements were resolved through discussion; when consensus could not be achieved, a third reviewer was consulted. This approach provided a structured and transparent evaluation of the internal validity of the included trials. Agreement between reviewers for risk-of-bias assessment was high (κ=0.84), with disagreements resolved through consensus.

### Data Extraction and Management

A standardized data extraction form was developed and pilot-tested for consistency. Further, 4 reviewers independently extracted data on study characteristics (authors, year, and country), sample size, participant age and gender, type of surgery, intervention type and duration, control condition, anxiety measurement tools, outcome data (mean scores, SDs, and *P* values), and parental anxiety or treatment adherence if reported. Extracted data were cross-checked and managed using Microsoft Excel (Microsoft Corp). Any discrepancies were resolved through group discussion. Interrater agreement for data extraction was high (κ=0.88), indicating strong consistency between reviewers. The standardized data extraction form used in this review was developed and pilot-tested for consistency. The full extraction template, including variable definitions, is provided in Multimedia Appendix 2 to enhance transparency and reproducibility.

### Statistical Analysis

All statistical analyses were conducted using R (R Foundation; version 4.3) with the *meta* and *metafor* packages. A random-effects meta-analysis model was used to account for expected clinical and methodological heterogeneity among the included studies. Studies were included in the meta-analysis only if they provided sufficient quantitative data to calculate standardized mean differences (SMDs; ie, means and SDs or equivalent statistics) at comparable preoperative time points, and used outcome measures considered sufficiently similar for pooling. Given the use of multiple validated anxiety measurement instruments across studies, effect sizes were standardized using SMDs with 95% CIs. While this approach allows pooling across different scales, it assumes that these instruments capture sufficiently comparable constructs of preoperative anxiety despite differences in measurement approach and underlying dimensions (Multimedia Appendix 3). Statistical heterogeneity was assessed using the *I*^2^ statistic and the Cochran Q test. An *I*^2^ value greater than 50% or a *P* value less than .10 from the Q test was considered indicative of substantial heterogeneity. Subgroup or sensitivity analyses were considered but ultimately not performed due to the small number of studies included in the meta-analysis (n=5). Conducting such analyses with a limited number of studies would result in low statistical power and a high risk of producing unstable or spurious findings. Therefore, these analyses were deemed methodologically inappropriate in this context. Publication bias assessment (eg, funnel plot or Egger test) was not performed because fewer than 10 studies were included in the pooled meta-analysis, limiting interpretability and statistical power [16]. All analyses were conducted with a significance level set at *P*<.05.

## Results

### Study Selection

**Figure 1:**
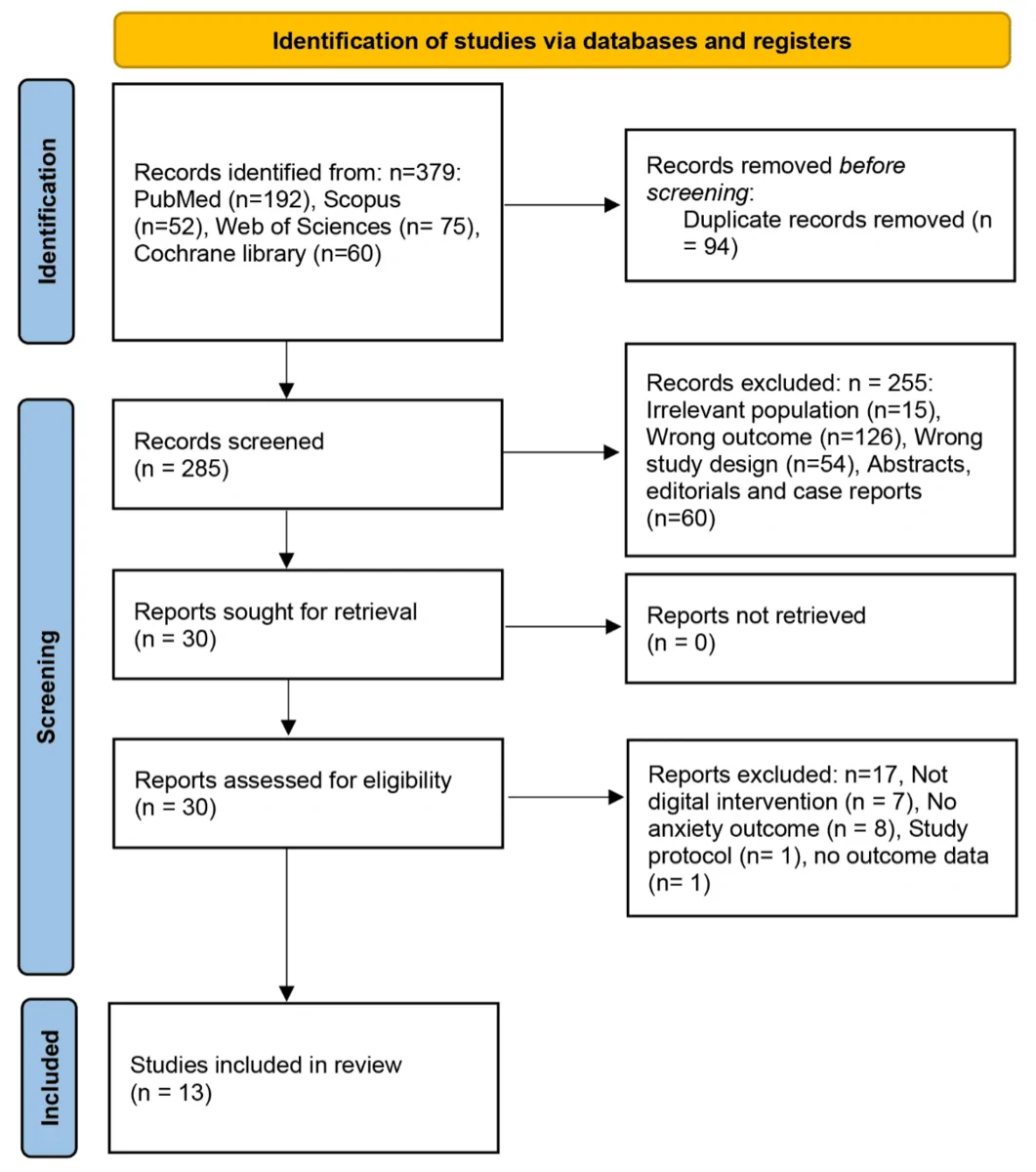
PRISMA flow diagram of study selection for the systematic review and meta-analysis. PRISMA: Preferred Reporting Items for Systematic Reviews and Meta-Analyses.

A total of 379 records were initially identified through database searches: PubMed (n=192), Scopus (n=52), Web of Science (n=75), and the Cochrane Library (n=60; Figure 1). After removing 94 duplicates, 285 unique records were screened by title and abstract. During this stage, 255 records were excluded due to irrelevant population (n=15); wrong outcome (n=126); inappropriate study design (n=54); or publication type such as abstracts, editorials, or case reports (n=60). A total of 30 full-text papers were assessed for eligibility. Of these, 17 studies were excluded, including studies evaluating nondigital interventions (n=7), studies not reporting anxiety outcomes (n=8), and 2 additional publications that were not eligible for synthesis (a protocol and a secondary evidence synthesis). Ultimately, 13 studies met the inclusion criteria and were included in the final systematic review.

### Characteristics of Included Studies

The 13 included studies were published between 2017 and 2025 and were conducted across multiple countries, including South Korea, Canada, Iran, the Netherlands, Turkey, China, Finland, and Spain (Table 1). Most studies were RCTs, with sample sizes ranging from 40 to 241 participants. The included studies primarily involved pediatric surgical populations, with some variability in reported age ranges, representing a potential source of clinical heterogeneity. The proportion of male participants varied across trials, including 1 study enrolling male-only participants (circumcision surgery). The types of surgery included general pediatric, ENT (ear, nose, and throat), ophthalmic, orthopedic, dental, abdominal, and circumcision procedures and were largely elective.

**Table 1:** Characteristics of included studies and participant demographics. Gender percentages refer to male distribution within each group when available. Surgeries are elective unless otherwise specified.

Study	Country	Design	Sample size (IG^a^/CG^b^)	Age range (y)	Male (IG/CG; %)^c^	Surgery type
Ryu et al, 2017 [17]	South Korea	RCT^d^	69 (34/35)	4-10 (6^e^)	50/68.6	Elective inpatient
Chow et al, 2017 [18]	Canada	Pilot RCT	100 (20/20)	7-13^f^	50-55	Outpatient ENT^g^ or herniorrhaphy
Park et al, 2019 [19]	South Korea	RCT	80 (40/40)	4-10^f^	68/50	Mixed elective
Ryu et al, 2019 [20]	South Korea	RCT	80 (41/39)	4-10 (6^e^)	71/54	ENT, orthopedic, dental
Dehghan et al, 2019 [21]	Iran	Solomon 4-group RCT	40 (20/20)	6-12^f^ (7.35)	77.5	Abdominal surgery
Eijlers et al, 2019 [22]	Netherlands	RCT	191 (94/97)	4-12 (8^e^)	48/58	ENT, dental, general
Buyuk et al, 2021 [23]	Turkey	RCT	78 (40/38)	5-10^f^	100 (male)	Circumcision
Wu et al, 2022 [24]	China	RCT	99 (51/48)	4-12 (7^e^)	86.3/87.5	ENT, hernia, penoplasty
Chamberlan d et al, 2024 [25]	Canada	RCT	101 (37/64)	5-17 (8-9^e^)	70/55	Mixed elective pediatric surgeries
Lee et al, 2023 [26]	South Korea	RCT	102 (51/51)	4-8^f^	54.9/47.1	Ophthalmic, ENT, others
Kerimaa et al, 2023 [27]	Finland	RCT	70 (36/34)	2-6^f^	Not reported	Mixed day surgery
Carbó et al, 2024 [28]	Spain	RCT	241 (120/121 )	3-12^f^ (6.7/6.2)	83.3/71.1	General pediatric surgeries
Pan et al, 2025 [29]	China	RCT	104 (52/52)	4-10^f^	65	ENT surgeries

^a^IG: intervention group.

^b^CG: control group.

^c^Gender percentages refer to male distribution within each group to clarify baseline demographics.

^d^RCT: randomized controlled trial.

^e^Median.

^f^Mean.

^g^ENT: ear, nose, and throat.

### Intervention Characteristics

Digital interventions varied in type and delivery mode across the included studies (Table 2). VR interventions were the most frequently evaluated modality and were typically delivered using head-mounted devices such as Samsung Gear VR, HTC Vive, Meta Quest 2, or NOLO VR systems. AR and MR approaches were less commonly studied and were delivered through AR-enabled devices, including platforms such as Microsoft HoloLens (Microsoft Corp). A smaller number of studies evaluated mobile or tablet-based apps, including digital storytelling interventions and video-based educational preparation tools.

**Table 2:** Intervention characteristics and control conditions. Narrative content indicates whether the digital intervention included a story-based structure, character guidance, or procedural walkthrough. Delivery platforms range from immersive headsets to mobile or tablet apps. Duration and timing reflect when and for how long the intervention was used relative to the surgery. Interventions marked as “not reported” were either not stated or unclear in the original studies.

Study	Digital tool type	Delivery mode and platform	Narrative content	Control condition	Timing	Duration (min)
Ryu et al, 2017 [17]	VR^a^ tour	Gear VR headset )Samsung)	Pororo cartoon guide	Verbal info	1 h preoperative	4
Chow et al, 2017 [18]	STM^b^ tablet app	Android tablet	Story-led 3D cartoon	Usual care	Home and preoperative day	20
Park et al, 2019 [19]	VR + parental mirroring	Gear VR + Smart Mirroring (Samsung)	Pororo cartoon	VR (child-only)	1 h preoperative	4
Ryu et al, 2019 [20]	VR tour	Gear VR (Samsung)	Pororo cartoon guide	Verbal info	1 h preoperative	4
Dehghan et al, 2019 [21]	VR exposure	Headset + PC	OR simulation	Usual care	Immediate preoperative	5
Eijlers et al, 2019 [22]	VR exposure	HTC Vive (HTC corporation)	Perioperative journey	Routine care	Preoperative day	15
Buyuk et al, 2021 [23]	VR distraction	VR BOX 3.000 headset	No narrative	Standard care	Preoperative	4.5
Wu et al, 2022 [24]	VR simulation	NOLO VR	Mascot “Tantan” narrates	Nonnarrative animation + verbal	1 d preoperative	5
Chamberland et al, 2024 [25]	AR^c^ + relaxation	Microsoft HoloLens 2	Story with characters	Standard care	Admission to OR^d^	≥20
Lee et al, 2023 [26]	3D VR education	Meta Quest 2	Characters explaining procedures	Tablet video	10 min preoperative	4
Kerimaa et al, 2023 [27]	Mobile app	BuddyCare (Buddy healthcare Ltd)	Story-based visuals/timeline	Routine education	Home and preoperative	Not reported
Carbó et al, 2024 [28]	VR education	Gear VR (Samsung)	Surgical journey walkthrough	Verbal/written info	7-10 d prior	≈5
Pan et al, 2025 [29]	Mobile app video	Smartphone QR code	Hospital staff explain the process	No app	1 d preoperative	11

^a^VR: virtual reality.

^b^STM: story-telling medicine.

^c^AR: augmented reality.

^d^OR: operating room.

Narrative elements, such as animated characters, guided procedural walkthroughs, and story-based visuals, were incorporated into many of the interventions, whereas a subset focused primarily on distraction without structured storytelling content. Control conditions generally consisted of standard preoperative care, including routine verbal and/or written information, usual education, or nonnarrative digital content. The timing and duration of interventions varied, ranging from immediate preoperative exposure to delivery up to 2 weeks before surgery, with most interventions lasting approximately 4 to 20 minutes.

### Effectiveness of Digital Interventions

Anxiety outcomes were assessed using a range of validated instruments, including observer-rated measures (eg, mYPAS or Modified Yale Preoperative Anxiety Scale Short Form, or Yale Preoperative Anxiety Scale) and self-report tools (eg, STAI, VAS, or Children’s Perioperative Multidimensional Anxiety Scale), across multiple perioperative time points. Table 3 provides a detailed summary of outcome measures, assessment timing, and between-group effects for each included study, along with reported findings on parental anxiety and intervention feasibility.

Across studies, most interventions were associated with lower levels of preoperative anxiety compared with control conditions, although the magnitude and statistical significance of these effects varied.

**Table 3:** Impact of digital interventions on preoperative anxiety, parental stress, and treatment compliance. “Reduced anxiety” indicates lower anxiety scores in the intervention group compared with the control group. “No significant between-group difference” indicates statistical nonsignificance between intervention and control at reported time points. Time points (T0-T6) are reported as defined in the original studies.

Study	Anxiety measures	Assessment time points	Effect on child preoperative anxiety (between-group comparison)	Parental anxiety	Feasibility or adherence
Ryu et al, 2017 [17]	mYPAS^a^; PBRS^b^; ICC^c^	30 min post	Significant reduction in anxiety in VR^d^ group (*P*<.001)	Not reported	Improved induction compliance (↑ ICC) and behavior (↓ PBRS)
Chow et al, 2017 [18]	CPMAS^e^	T1-T3	Significant reduction in anxiety in digital storytelling group (large effect size; Cohen *d*=0.96)	Not reported	90% completed the intervention
Park et al, 2019 [19]	mYPAS; NRS^f^	Baseline; induction	Significant reduction in anxiety in the mirrored VR group (*P*=.025)	Significant reduction in parental anxiety (NRS; *P*=.009)	Comparable adherence reported
Ryu et al, 2019 [20]	mYPAS	Baseline; preinduction	Significant reduction in anxiety in the VR group (*P*<.05)	Not reported	Not reported
Dehghan et al, 2019 [21]	YPAS^g^	Pre or post	Significant reduction in anxiety in the VR group (*P*<.05)	Not reported	Not reported
Eijlers et al, 2019 [22]	mYPAS; VAS^h^	T1-T5	No significant between-group difference	No significant between-group difference (STAI-S^i^)	22% removed VR headset
Buyuk et al, 2021 [23]	CAM-S^j^	Pre or post	Significant reduction in anxiety in the VR group (*P*<.001)	Not reported	100% completed intervention
Wu et al, 2022 [24]	mYPAS-SF^k^	T1-T5	Significant reduction in anxiety in VR group at specific time points (eg, T3; *P*=9 × 10⁻⁶)	No significant between-group difference (STAI^l^)	Improved induction compliance (↑ ICC; *P*=.003)
Chamberland et al, 2024 [25]	mYPAS-SF	Admission; induction	Significant reduction in anxiety in the AR^m^ group compared with the control	Not reported	Dropouts reported (n=19 in AR group)
Lee et al, 2023 [26]	mYPAS	Baseline; preinduction	Significant reduction in anxiety in VR compared with tablet video (*P*=.02)	Not reported	Better induction compliance in VR (ICC; *P*=.007)
Kerimaa et al, 2023 [27]	VAS; STAI-S; FAS^n^	T1-T4	No significant between-group difference	No significant between-group difference	100% completed intervention
Carbó et al, 2024 [28]	mYPAS/m YPAS-SF	T0-T2	Significant reduction in anxiety in the VR education group (*P*<.001)	No significant between-group difference (STAI-S)	100% completed intervention
Pan et al, 2025 [29]	mYPAS-SF; VAS	T0-T4	Significant reduction in anxiety in the mobile-based intervention group (*P*=.012)	Significant reduction in parental anxiety (VAS; *P*=.013)	100% completed intervention

^a^mYPAS: Modified Yale Preoperative Anxiety Scale.

^b^PBRS: Procedure Behavior Rating Scale.

^c^ICC: Induction Compliance Checklist.

^d^VR: virtual reality.

^e^CPMAS: Children’s Perioperative Multidimensional Anxiety Scale.

^f^NRS: Numeric Rating Scale.

^g^YPAS: Yale Preoperative Anxiety Scale.

^h^VAS: Visual Analog Scale.

^i^STAI-S: state subscale of State-Trait Anxiety Inventory.

^j^CAM-S: Children’s Anxiety Meter Scale.

^k^mYPAS-SF: Modified Yale Preoperative Anxiety Scale Short Form.

^l^STAI: State-Trait Anxiety Inventory.

^m^AR: augmented reality.

^n^FAS: Facial Affective Scale.

To provide a more systematic overview of findings, a structured narrative synthesis was conducted to assess the direction and consistency of intervention effects across all included studies (Table 4). Direction of effect was categorized as reduction in anxiety, no difference, or unclear, and statistical significance was determined based on between-group comparisons. As shown in Table 4, most studies (11 of 13) reported reductions in preoperative anxiety in the intervention groups compared with standard care or control conditions, with most demonstrating statistically significant between-group differences.

**Table 4:** Summary of direction and statistical significance of intervention effects across included studies.

Study	Intervention type	Anxiety outcome	Direction of effect	Statistical significance
Ryu et al, 2017 [17]	VR^a^	mYPAS^b^	↓ Anxiety	Significant
Chow et al, 2017 [18]	Digital storytelling	CPMAS^c^	↓ Anxiety	Significant
Park et al, 2019 [19]	VR + parental mirroring	mYPAS	↓ Anxiety	Significant
Ryu et al, 2019 [20]	VR	mYPAS	↓ Anxiety	Significant
Dehghan et al, 2019 [21]	VR	YPAS^d^	↓ Anxiety	Significant
Eijlers et al, 2019 [22]	VR	mYPAS, VAS^e^	No clear difference	Not significant
Buyuk et al, 2021 [23]	VR	CAM-S^f^	↓ Anxiety	Significant
Wu et al, 2022 [24]	VR	mYPAS-SF^g^	↓ Anxiety	Significant
Chamberland et al, 2024 [25]	AR^h^	mYPAS-SF	↓ Anxiety	Significant
Lee et al, 2023 [26]	VR vs tablet	mYPAS	↓ Anxiety	Significant
Kerimaa et al, 2023 [27]	Mobile app	VAS, STAI^i^	No clear difference	Not significant
Carbó et al, 2024 [28]	VR	mYPAS	↓ Anxiety	Significant
Pan et al, 2025 [29]	Mobile app	mYPAS-SF, VAS	↓ Anxiety	Significant

^a^VR: virtual reality.

^b^mYPAS: Modified Yale Preoperative Anxiety Scale.

^c^CPMAS: Children’s Perioperative Multidimensional Anxiety Scale.

^d^YPAS: Yale Preoperative Anxiety Scale.

^e^VAS: Visual Analog Scale.

^f^CAM-S: Children’s Anxiety Meter Scale.

^g^mYPAS-SF: Modified Yale Preoperative Anxiety Scale Short Form.

^h^AR: augmented reality.

^i^STAI: State-Trait Anxiety Inventory.

A total of 2 studies (Eijlers et al [22]; Kerimaa et al [27]) did not report statistically significant between-group differences, although both observed reductions in anxiety over time within groups. Overall, these findings indicate a generally consistent direction of effect favoring digital interventions, with variability in effect size and statistical significance likely reflecting differences in intervention type, timing of delivery, population characteristics, and outcome measurement tools. This structured synthesis complements the meta-analysis by highlighting patterns of consistency despite substantial heterogeneity.

### Meta-Analysis Findings

**Figure 2:**
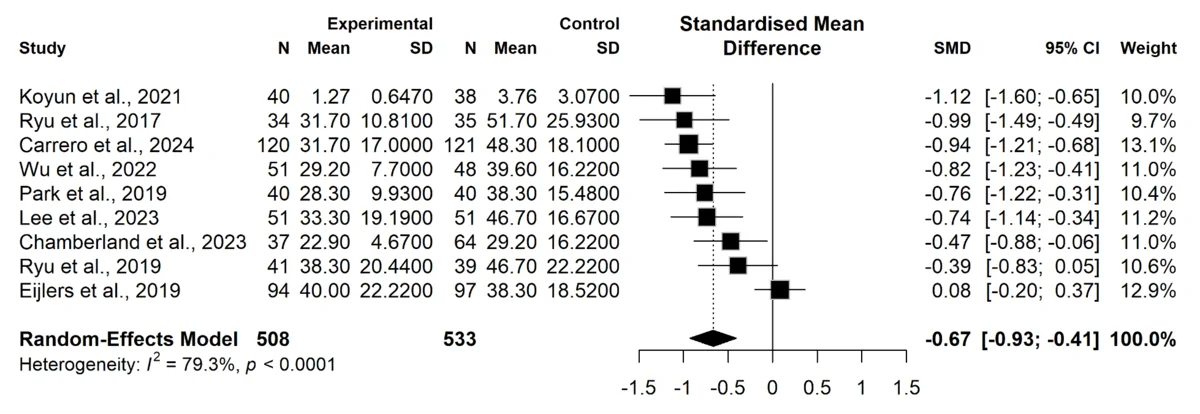
Forest plot showing the effectiveness of digital storytelling and immersive technologies in reducing preoperative anxiety among children.

Figure 2 [17,19,20,22-26,28] presents the results of the random-effects meta-analysis evaluating the effects of digital interventions on preoperative anxiety in pediatric patients undergoing elective surgery. Nine studies comprising 508 participants in the intervention groups and 533 participants in the control groups were included. Most studies demonstrated lower anxiety scores in children receiving digital interventions, with SMDs ranging from −1.12 to 0.08. The pooled analysis showed a statistically significant reduction in preoperative anxiety favoring digital interventions compared with standard care or conventional preparation methods (SMD=−0.67, 95% CI −0.93 to −0.41). However, substantial heterogeneity was observed across studies (*I*^2^=79.3%, *P*<.001), indicating considerable variability in effect sizes. Given this heterogeneity and the diversity of intervention types, populations, and outcome measures, the pooled estimate should be interpreted with caution. Nevertheless, the overall direction of effect across studies generally favored digital interventions for reducing preoperative anxiety.

### Risk of Bias Assessment

**Figure 3:**
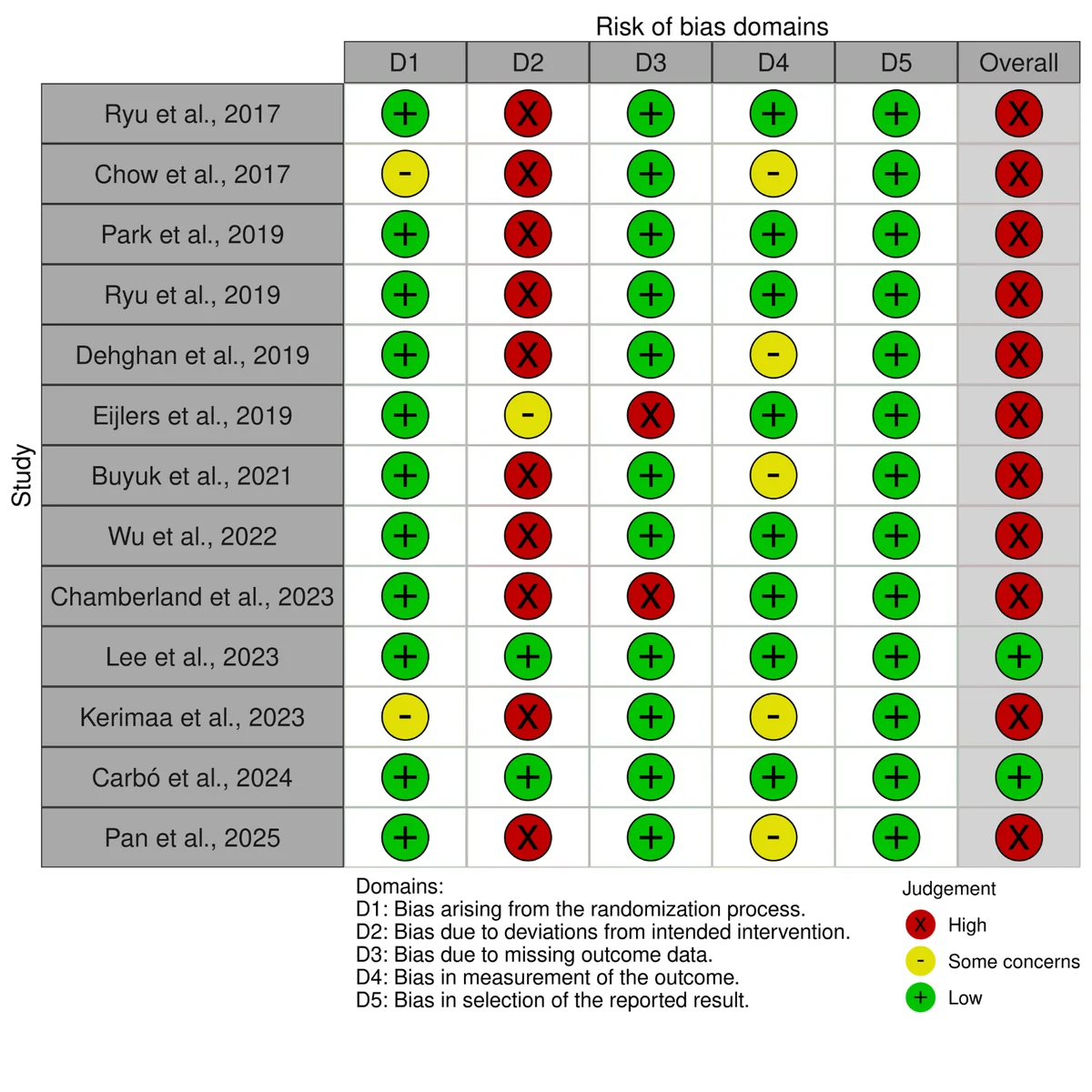
Risk of bias assessment of included studies.

Risk of bias assessments using RoB 2 are summarized in Figure 3 [17-29]. For bias arising from the randomization process (D1), most studies were judged to be at low risk, while a minority raised some concerns due to limited reporting of allocation procedures. Bias due to deviations from intended interventions (D2) was the most frequent limitation, as blinding of participants and personnel was generally not feasible for interactive interventions (eg, VR or AR or app-based tools), potentially influencing engagement and subjective anxiety reporting.

Most studies were judged at low risk for missing outcome data (D3), although a small number had high risk or some concerns due to attrition or incomplete reporting. Bias in measurement of the outcome (D4) and selection of the reported result (D5) was generally low, with some concerns in studies with insufficient reporting detail. Overall, 2 studies were judged as low risk of bias (Carbó et al [28]; Lee et al [26]), 1 study had some concerns, and the remaining trials were judged as high risk of bias, largely driven by D2 (deviations from intended interventions).

## Discussion

### Principal Findings

This systematic review and meta-analysis synthesized the available evidence on the effectiveness of digital storytelling and immersive technology for reducing preoperative anxiety in children.

The findings suggest that digital storytelling and immersive technologies may reduce preoperative anxiety in children, although interpretation should remain cautious given heterogeneity and the overall risk of bias across included trials. These results align with a growing body of literature advocating for nonpharmacological, child-centered approaches to improve the perioperative experience.

The significant anxiety reduction observed in our analysis (SMD=–0.99) strongly supports the efficacy of digital interventions and can be understood through 2 primary psychological mechanisms: cognitive distraction and cognitive remodeling. These findings are consistent with a robust and growing body of evidence demonstrating the power of technology to positively shape the pediatric perioperative experience [30,31], the substantial heterogeneity observed (*I*^2^=78.1%), and the small number of studies included in the meta-analysis limit the interpretability and clinical applicability of the pooled estimate.

This heterogeneity likely reflects multiple sources of variability across studies. First, interventions differed in modality and underlying mechanisms, including immersive technologies (eg, VR and AR) and narrative-based digital storytelling applications, which may influence anxiety through distinct pathways such as distraction, familiarization, or cognitive restructuring. Second, there was variation in the timing and duration of intervention delivery, ranging from immediate preoperative exposure to preparation delivered several days before surgery. Third, differences in clinical populations, including age ranges, types of surgical procedures, and perioperative contexts, may have influenced baseline anxiety levels and responsiveness to intervention. Finally, variability in outcome measurement, particularly the use of both observer-rated tools (eg, mYPAS) and self-report instruments (eg, STAI and VAS), may contribute to differences in effect size estimation, as these instruments capture different dimensions of anxiety.

Given these sources of heterogeneity and the limited number of studies available for quantitative synthesis, the pooled effect estimate should be interpreted as an approximate indication of overall effect direction rather than a precise or generalizable measure of intervention efficacy.

Only 5 studies provided sufficiently comparable outcome data for meta-analysis, which further limits the robustness of the pooled estimate. The remaining studies were synthesized narratively and generally demonstrated reductions in anxiety, although differences in reporting formats and assessment time points restricted quantitative integration. Accordingly, greater emphasis should be placed on the consistency and direction of findings across individual studies rather than the pooled estimate alone.

While the included studies did not directly assess underlying psychological or neurophysiological mechanisms, several theoretical frameworks may help explain the observed reductions in preoperative anxiety. One possible explanation, particularly for immersive technologies such as VR, is cognitive distraction. Grounded in the limited capacity model of attention, this principle suggests that the brain has finite resources to process simultaneous stimuli [10]. By immersing a child in a compelling, interactive, and sensorially rich virtual environment, these interventions may engage attentional resources, thereby reducing the capacity available to process anxiogenic cues from the clinical setting, such as unfamiliar equipment or procedural environments [32].

Evidence from prior research outside the included trials suggests that immersive environments may influence neural processing related to pain and emotional distress, including activity in regions such as the thalamus, insula, and anterior cingulate cortex [33]. In addition, the sense of “presence”—the subjective feeling of being immersed in a virtual environment—may further enhance attentional engagement and contribute to distraction effects [34]. Similarly, cognitive-behavioral frameworks support the role of gradual exposure and improved understanding in reducing anxiety responses. However, it is important to emphasize that these mechanisms were not directly measured in the included studies, which primarily assessed anxiety using behavioral or self-report instruments. Therefore, these explanations should be interpreted as theoretical rather than empirically established within this review. Additionally, different intervention types (eg, VR, AR, and narrative-based digital applications) are likely to operate through distinct mechanisms, and applying a single explanatory framework across all modalities may oversimplify their effects.

Our findings are consistent with existing systematic reviews and meta-analyses showing that immersive digital interventions, particularly VR, can reduce perioperative anxiety in pediatric populations. For example, Chen et al [30] conducted a systematic review and meta-analysis of randomized trials and reported that VR-based preoperative preparation reduced anxiety and improved induction compliance in children. Similarly, Eijlers et al [31] synthesized pediatric evidence across medical procedures and concluded that VR is an effective nonpharmacological intervention for reducing anxiety and pain in children. Other meta-analyses focused on surgical contexts have also reported beneficial effects of preoperative VR on anxiety, supporting the direction of effect observed in the present review [35]. In addition, more recent reviews have highlighted the growing interest in immersive perioperative tools, including VR and emerging AR platforms, although the evidence base remains heterogeneous and varies in methodological quality [8]. Taken together, our findings reinforce prior evidence supporting VR-based preparation, while extending the literature by incorporating newer formats such as digital storytelling and app-based interventions alongside immersive technologies.

Beyond distraction, several interventions, particularly those providing guided procedural walkthroughs or narrative-based preparation (eg, Wu et al [24]), may promote cognitive restructuring through procedural familiarization and skills rehearsal [24]. These tools function as a form of digital exposure therapy or guided preparation, allowing children to “rehearse” the surgical journey in a safe and engaging format [8]. By following a relatable animated character or interacting with a virtual model of the operating room, a child’s fear of the unknown is replaced with predictability and understanding [8]. This process aligns with principles of cognitive-behavioral therapy, where demystifying a stressful event helps to correct maladaptive thoughts and reduce anticipatory anxiety [36]. Furthermore, the interactive nature of these tools can instill a sense of agency and control in children, directly counteracting the feelings of powerlessness that often fuel preoperative anxiety.

This dual-action approach helps explain why these digital interventions often outperform traditional nonpharmacological methods [37]. While printed materials are static and verbal explanations can be difficult for young, distressed children to process, a digital narrative is dynamic and developmentally appropriate [8]. This is especially true for younger children, who are more receptive to narrative-based learning and “magical thinking,” making them ideal candidates for story-based interventions [38]. Ultimately, the interventions included in our review represent a sophisticated evolution of patient preparation, leveraging technology to create experiences that are not only distracting but also educational and empowering, leading to the substantial reductions in anxiety observed in our meta-analysis [39].

### Limitations

Despite the promising findings, several limitations should be considered when interpreting this review. First, the overall certainty of evidence was limited by risk of bias, with most trials judged as high risk, particularly in the RoB 2 domain related to deviations from intended interventions, as blinding of participants and personnel was not feasible for interactive digital tools (eg, VR or AR and app-based preparation). This lack of blinding may have introduced performance bias, potentially inflating the observed intervention effects, especially for subjective anxiety outcomes [28].

Second, substantial clinical and methodological heterogeneity was present across studies, including differences in intervention type and duration, surgical settings, timing of delivery, and outcome assessment points. This heterogeneity represents a key source of uncertainty and limits the interpretability and generalizability of the pooled findings. In particular, variability in intervention modality (eg, VR, AR, and digital storytelling), differences in delivery timing (immediate vs days before surgery), and diversity in patient populations (age ranges and surgical types) may have contributed to the observed inconsistency in effect sizes.

An additional limitation relates to the heterogeneity of outcome measures. The included studies used a range of anxiety instruments, including observer-rated tools (eg, mYPAS) and self-report measures (eg, STAI or VAS), which capture different dimensions of anxiety (behavioral vs subjective). Although the use of SMDs enables statistical pooling across scales, it does not fully address conceptual differences between instruments. These differences may result in nonequivalent outcome constructs being combined, thereby contributing to statistical heterogeneity and uncertainty in the pooled estimate. Therefore, the pooled estimate should be interpreted with caution, as it may combine outcomes that are not entirely equivalent.

Third, only a subset of studies provided sufficient and comparable outcome data for meta-analysis, and most eligible studies were synthesized narratively, which may reduce the precision of pooled estimates. The small number of studies included in the meta-analysis (n=5) also limited the feasibility of conducting subgroup or sensitivity analyses, restricting further exploration of potential sources of heterogeneity. Publication bias could not be reliably assessed because fewer than 10 studies were included in the quantitative synthesis.

Finally, some trials included broader pediatric age ranges than initially planned, and relatively few studies assessed secondary outcomes such as parental anxiety, adherence, postoperative behavioral changes, or longer-term recovery, limiting conclusions regarding the wider impact of these interventions on families and perioperative outcomes. Taken together, these limitations indicate that the findings should be interpreted as indicative rather than definitive, and highlight the need for more standardized, high-quality studies to strengthen the evidence base.

### Implications for Practice and Research

The results of this review suggest that immersive and storytelling-based technologies represent a feasible, engaging, and effective adjunct to conventional preoperative preparation. Their scalability and adaptability across cultural and clinical settings make them attractive options in pediatric care. To strengthen the evidence base, future trials should focus on improving methodological rigor, particularly with respect to blinding and standardized reporting.

Implementation feasibility may differ across contexts. In resource-rich perioperative units, VR or AR headsets may offer strong engagement but require equipment, staff support, and infection-control processes. In lower-resource settings or high-throughput outpatient clinics, tablet-based storytelling apps or brief mobile interventions may represent more practical and scalable alternatives. Further research should also investigate cost-effectiveness, age-specific preferences, and the differential value of narrative vs nonnarrative content. Importantly, studies should extend beyond immediate preoperative anxiety to examine outcomes such as postoperative behavior, recovery, and parental well-being, which are critical to holistic perioperative care.

### Conclusions

This systematic review and meta-analysis synthesized and critically appraised the available evidence on the effects of digital storytelling and immersive technology–based interventions on preoperative anxiety in pediatric patients undergoing elective surgery. Overall, the included studies suggest a trend toward reduced preoperative anxiety in children receiving digital interventions compared with standard care. However, the certainty of this evidence is limited. The pooled meta-analytic estimate should be interpreted with considerable caution due to substantial heterogeneity, the small number of studies included in the quantitative synthesis, and the overall risk of bias across trials. In addition, variability in intervention types (eg, VR, AR, and narrative-based applications), outcome measures, and study designs limits the ability to draw definitive or generalizable conclusions. While these interventions appear to be feasible and engaging approaches to preoperative preparation, current evidence does not allow firm conclusions regarding their comparative effectiveness or optimal implementation. Future high-quality studies with standardized outcome measures, clearly defined intervention protocols, and robust methodological design are needed. Further research should also examine parental anxiety, cost-effectiveness, and longer-term postoperative outcomes to better inform clinical practice.

## Data Availability

The datasets used and analyzed during this study are available from the corresponding author on reasonable request.

## Appendix

Multimedia Appendix 1 Search strategies.

Multimedia Appendix 2 Data extraction table.

Multimedia Appendix 3 Tables analyzing data on studies.

Multimedia Appendix 4 PRISMA 2020 checklist.

## Abbreviations

AR: augmented reality
ENT: ear, nose, and throat
MR: mixed reality
mYPAS: Modified Yale Preoperative Anxiety Scale
PICOS: Population, Intervention, Comparison, Outcomes, and Study
PRISMA: Preferred Reporting Items for Systematic Reviews and Meta-Analyses
RCT: randomized controlled trial
RoB 2: Cochrane Risk of Bias tool version 2
SMD: standardized mean difference
STAI: State-Trait Anxiety Inventory
VAS: Visual Analog Scale
VR: virtual reality
