# Supplementary material for: Effectiveness of Digital Storytelling and Immersive Technology Interventions in Reducing Preoperative Anxiety Among Children: Systematic Review and Meta-Analysis
Source: JMIR Pediatr Parent. 2026 Aug 5;9:e92880. doi: 10.2196/92880 (PMC13490931; doi:10.2196/92880)
Supplement: Supplementary file 2 [file pediatrics_v9i1e92880_app1.docx]

**Search strategy for PubMed**

(child* OR pediatric* OR paediatric*)

AND

(preoperative anxiety OR surgical anxiety OR anxiety reduction OR procedural anxiety)

AND

(storytelling OR "digital storytelling" OR "interactive story" OR "animated book" OR "augmented reality" OR "AR book" OR "virtual reality" OR VR OR "tablet application" OR "mobile app" OR "educational video" OR "video-based education")

AND

(surgery OR "surgical preparation" OR "hospital preparation")

**Search strategy for Scopus**

(TITLE-ABS-KEY(child* OR pediatric* OR paediatric*) AND

TITLE-ABS-KEY("preoperative anxiety" OR "surgical anxiety" OR "procedure-related anxiety" OR "hospital anxiety") AND

TITLE-ABS-KEY("digital storytelling" OR "interactive story" OR "animated book" OR "augmented reality" OR "AR book" OR "virtual reality" OR "VR" OR "tablet app*" OR "mobile app*" OR "video-based education") AND

TITLE-ABS-KEY(surgery OR "surgical preparation" OR "hospital preparation"))

**Search strategy for Web of Science (WoS)**

TS=(child* OR pediatric* OR paediatric*) AND

TS=("preoperative anxiety" OR "surgical anxiety" OR "hospital anxiety" OR "procedure-related anxiety") AND

TS=("digital storytelling" OR "interactive story" OR "animated book" OR "augmented reality" OR "AR book" OR "virtual reality" OR "VR" OR "mobile app*" OR "tablet app*" OR "video-based education") AND

TS=(surgery OR "surgical preparation" OR "hospital preparation")

**Search strategy for Cochrane Library**

(child* OR pediatric* OR paediatric*) AND

("preoperative anxiety" OR "surgical anxiety" OR "procedure-related anxiety") AND

("digital storytelling" OR "interactive story" OR "animated storybook" OR "augmented reality" OR "virtual reality" OR "VR" OR "mobile app*" OR "tablet app*" OR "video education") AND

(surgery OR "hospital preparation")
