# Supplementary material for: Effectiveness of Digital Storytelling and Immersive Technology Interventions in Reducing Preoperative Anxiety Among Children: Systematic Review and Meta-Analysis
Source: JMIR Pediatr Parent. 2026 Aug 5;9:e92880. doi: 10.2196/92880 (PMC13490931; doi:10.2196/92880)
Supplement: Supplementary file 3 [file pediatrics_v9i1e92880_app2.docx]

**Table S1.** Standardized data extraction template.

| **Section** | **Variable** | **Description / Definition** | **Data Type / Format** |
| --- | --- | --- | --- |
| **Study identification** | Author(s) | First author and year of publication | Text |
|  | Country | Country where study was conducted | Text |
|  | Study design | RCT, quasi-experimental, etc. | Categorical |
| **Participants** | Sample size (IG/CG) | Number of participants in intervention and control groups | Numeric |
|  | Age | Age range and/or mean/median age | Numeric / Text |
|  | Gender | Percentage or number of male participants | Numeric (%) |
|  | Inclusion criteria | Key inclusion criteria reported in study | Text |
|  | Exclusion criteria | Key exclusion criteria reported in study | Text |
| **Clinical context** | Type of surgery | Surgical procedure(s) (e.g., ENT, dental, general) | Text |
|  | Setting | Inpatient, outpatient, day surgery, etc. | Text |
| **Intervention** | Intervention type | VR, AR, digital storytelling, mobile app, etc. | Categorical |
|  | Intervention description | Detailed description of content (e.g., narrative, simulation) | Text |
|  | Delivery mode | Device/platform used (e.g., headset, tablet) | Text |
|  | Timing | When intervention was delivered (e.g., 1 hour pre-op) | Text |
|  | Duration | Length of intervention exposure | Numeric / Text |
| **Comparator** | Control condition | Description of standard care or comparator intervention | Text |
| **Outcomes** | Anxiety measure(s) | Instrument(s) used (e.g., mYPAS, STAI, VAS) | Text |
|  | Outcome type | Observer-rated or self-report | Categorical |
|  | Assessment time point(s) | Timing of outcome measurement (e.g., baseline, induction) | Text |
|  | Outcome data | Means, standard deviations, or equivalent statistics | Numeric |
| **Secondary outcomes** | Parental anxiety | Instrument and results if reported | Text |
|  | Feasibility / adherence | Completion rates, dropouts, usability issues | Text |
| **Risk of bias** | RoB 2 domains | D1–D5 judgments (low/some concerns/high) | Categorical |
|  | Overall risk of bias | Overall study judgment | Categorical |
| **Additional notes** | Comments | Any relevant notes or clarifications | Text |
