# Supplementary material for: Effectiveness of Digital Storytelling and Immersive Technology Interventions in Reducing Preoperative Anxiety Among Children: Systematic Review and Meta-Analysis
Source: JMIR Pediatr Parent. 2026 Aug 5;9:e92880. doi: 10.2196/92880 (PMC13490931; doi:10.2196/92880)
Supplement: Supplementary file 4 [file pediatrics_v9i1e92880_app3.docx]

**Table S1.** Characteristics of anxiety measurement instruments used in included studies.

| **Instrument** | **Type of assessment** | **Primary construct measured** | **Informant** | **Typical application in included studies** |
| --- | --- | --- | --- | --- |
| mYPAS / mYPAS-SF | Observer-rated | Behavioral manifestations of state anxiety | Trained observer | Preoperative observation (e.g., holding area, induction) |
| YPAS | Observer-rated | Behavioral anxiety responses | Trained observer | Pre/post intervention behavioral assessment |
| STAI / STAI-S | Self-report | Subjective state anxiety (cognitive/emotional) | Child or parent (depending on study) | Preoperative anxiety assessment, often in older children or caregivers |
| VAS | Self-report | Perceived anxiety intensity (single-item scale) | Child or parent | Rapid assessment of anxiety at specific time points |
| CPMAS | Self-report | Multidimensional perioperative anxiety (cognitive, emotional, physiological) | Child | Preoperative assessment across multiple domains |
| CAM-S | Self-report | State anxiety (pictorial/scale-based) | Child | Pre/post intervention comparison, often in younger children |
| FAS | Self-report (visual scale) | Emotional distress / affective state | Child | Simple visual assessment in younger children |

These instruments differ in terms of respondent (observer vs self-report), dimensionality (single-item vs multidimensional), and the specific aspects of anxiety assessed (behavioral, cognitive, or affective). This variability should be considered when interpreting pooled estimates.

**Table S2.** Availability of outcome data and reasons for inclusion or exclusion from meta-analysis.

| **Study** | **Anxiety outcome(s) reported** | **Sufficient data for SMD (mean/SD or equivalent)** | **Comparable preoperative time point** | **Included in meta-analysis** | **Reason for exclusion (if not included)** |
| --- | --- | --- | --- | --- | --- |
| Ryu et al., 2017 [17] | mYPAS | Yes | Yes | Yes | — |
| Chow et al., 2017 [18] | CPMAS | No | No | No | Insufficient quantitative data (means/SDs not reported) |
| Park et al., 2019 [19] | mYPAS | Yes | Yes | Yes | — |
| Ryu et al., 2019 [20] | mYPAS | Yes | Yes | Yes | — |
| Dehghan et al., 2019 [21] | YPAS | Yes | Yes | Yes | — |
| Eijlers et al., 2019 [22] | mYPAS; VAS | No | No | No | Multiple time points without extractable comparable data |
| Koyun et al., 2021 [23] | CAM-S | No | No | No | Outcome not directly comparable / insufficient statistical reporting |
| Wu et al., 2022 [24] | mYPAS-SF | Yes | No | No | Outcome reported at non-comparable time points |
| Chamberland et al., 2023 [25] | mYPAS-SF | No | No | No | Incomplete reporting of quantitative data |
| Lee et al., 2023 [26] | mYPAS | Yes | Yes | Yes | — |
| Kerimaa et al., 2023 [27] | VAS; STAI-S; FAS | No | No | No | Multiple instruments with no extractable pooled data |
| Carrero et al., 2024 [28] | mYPAS / mYPAS-SF | No | No | No | Insufficient reporting for effect size calculation |
| Pan et al., 2025 [29] | mYPAS-SF; VAS | No | No | No | Incomplete statistical reporting |

**Note:** Studies were included in the meta-analysis only if they reported sufficient quantitative data (e.g., means and standard deviations or equivalent statistics) at comparable preoperative time points and used outcome measures considered suitable for pooling. Studies were excluded when data were incomplete, reported at non-comparable time points, or when outcomes could not be meaningfully aligned.
